# Epigenetic silencing of miR-200b is associated with cisplatin resistance in bladder cancer

**DOI:** 10.18632/oncotarget.25326

**Published:** 2018-05-11

**Authors:** Tetsuya Shindo, Takeshi Niinuma, Naotaka Nishiyama, Nobuo Shinkai, Hiroshi Kitajima, Masahiro Kai, Reo Maruyama, Takashi Tokino, Naoya Masumori, Hiromu Suzuki

**Affiliations:** ^1^ Department of Urology, Sapporo Medical University School of Medicine, Sapporo 060-8543, Japan; ^2^ Department of Molecular Biology, Sapporo Medical University School of Medicine, Sapporo 060-8556, Japan; ^3^ Project for Cancer Epigenome, The Cancer Institute, Japanese Foundation for Cancer, Koto-ku, Tokyo 135-8550, Japan; ^4^ Medical Genome Science, Research Institute for Frontier Medicine, Sapporo Medical University School of Medicine, Sapporo 060-8556, Japan

**Keywords:** bladder cancer, cisplatin resistance, miRNA, DNA methylation, histone modification

## Abstract

In this study, we identified microRNAs (miRNAs) involved in cisplatin (CDDP) resistance in bladder cancer (BCa). After establishing CDDP-resistant BCa cell lines (T24RC and EJ138RC), TaqMan arrays revealed that members of the miR-200 family (miR-200b, miR-200a and miR-429) were downregulated in T24RC as compared to parental T24 cells. miR-200b was associated with CDDP sensitivity in BCa cells, and its downregulation was associated with CpG island hypermethylation. Pharmacological demethylation using 5-aza-2’-deoxycytidine restored miR-200b expression, and the combination of 5-aza-2’-deoxycytidine + CDDP strongly inhibited T24RC cell proliferation. Microarray analysis revealed that miR-200b + CDDP induced genes involved in CDDP sensitivity or cytotoxicity, including IGFBP3, ICAM1 and TNFSF10, in the resistant cells. Expression and DNA methylation of miR-200b were inversely associated in primary BCa, and low expression/high methylation was associated with poor overall survival. These results suggest downregulation of miR-200b is associated with CDDP resistance in BCa. Epigenetic silencing of miR-200b may be a marker of CDDP resistance and a useful therapeutic target for overcoming CDDP resistance in BCa.

## INTRODUCTION

Around 430,000 bladder cancers (BCas) were newly diagnosed worldwide in 2012 [1]. Approximately 70% of BCa is diagnosed as non-muscle invasive BCa (NMIBC), while the remaining 30% is muscle invasive (MIBC) [2]. For decades, the standard treatment for metastatic BCa has been cisplatin (CDDP)-based chemotherapy, though the survival period for patients treated with CDDP plus gemcitabine (GEM) is only 13.8 months [3, 4]. Resistance to CDDP is the major cause of treatment failure. CDDP is also widely used for neoadjuvant chemotherapy in MIBC, but the survival benefit is relatively small, as CDDP-based neoadjuvant chemotherapy reportedly provides only a 5% absolute improvement in survival at 5 years [5]. That said, a recent meta-analysis suggested that a pathological complete response to adjuvant chemotherapy is associated with better survival of MIBC patients [6]. Understanding the mechanism underlying chemoresistance would contribute to overcoming it and enable better identification of patients who will benefit from adjuvant chemotherapy.

MicroRNAs (miRNAs) are small (21–25 nucleotides) noncoding RNAs that act post-transcriptionally to suppress gene expression, and dysregulation of miRNAs has been implicated in the pathogenesis of BCa [7]. We previously reported that a number of miRNAs, including miR-137 and miR-124, are silenced in association with DNA hypermethylation of upstream CpG islands in BCa cells, and that epigenetic silencing of miRNAs may be associated with the development of BCa [8]. Recent studies also suggest the potential involvement of miRNAs in determining chemosensitivity. For instance, miR-34a is a well-known tumor suppressor that acts as a downstream effector of p53, and its induction reportedly sensitizes BCa cells to CDDP [9]. miR-34a is frequently downregulated by DNA methylation in MIBC, whereas CDDP induces DNA demethylation and transcriptional activation of miR-34a in BCa cells, which could lead to increased chemosensitivity [10]. In addition, miR-193a-3p reportedly promotes multi-chemoresistance by targeting multiple genes, including SRSF2, PLAU and HIC2 [11]. Moreover, decreased expression of miR-203 was shown to be an indicator of poor prognosis of BCa, while induction of miR-203 enhances the CDDP sensitivity of BCa cells [12].

To better understand the contribution made by miRNAs to acquired CDDP resistance in BCa, we established CDDP-resistant BCa cell lines and comprehensively analyzed their miRNA expression profiles. We found that a number of miRNAs were differentially expressed in BCa cells before and after acquisition of chemoresistance. Among the identified miRNAs, we found that epigenetic downregulation of miR-200b is associated with CDDP resistance, and that it could serve as a prognostic marker and as a therapeutic target to overcome chemoresistance.

## RESULTS

### Downregulation of miR-200b is associated with CDDP resistance in BCa cells

**Figure 1:**
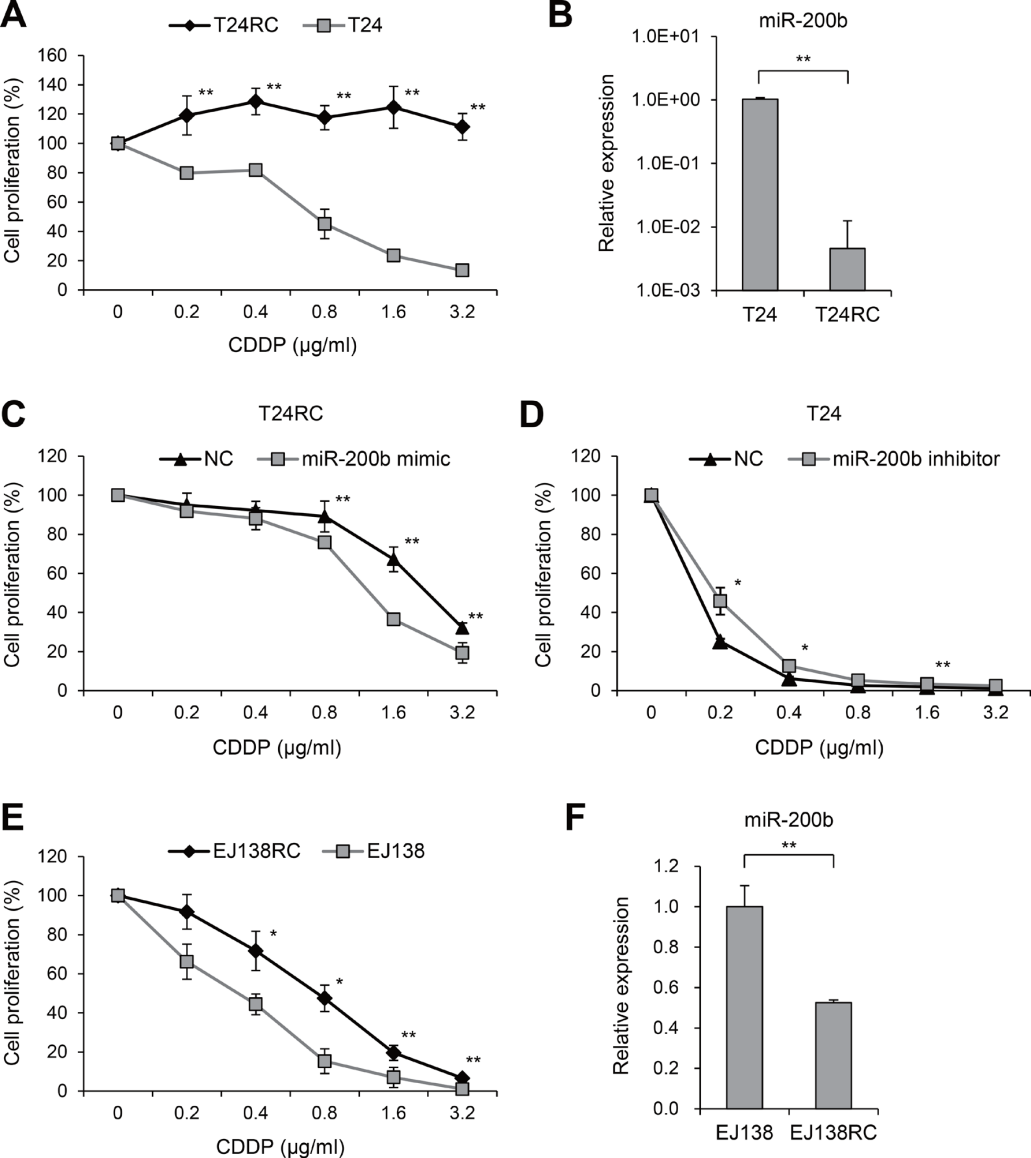
Downregulation of miR-200b in CDDP-resistant BCa cells (**A**) Anti-tumor effects of CDDP in T24 and T24RC cells. Numbers of cells treated with the indicated concentrations of CDDP are shown relative to those without treatment. (**B**) qRT-PCR analysis of miR-200b in T24 and T24RC cells. (**C**) Effects of miR-200b on CDDP sensitivity in T24RC cells. Numbers of cells transfected with a miR-200b mimic or negative control (NC) and then treated with the indicated concentrations of CDDP are shown relative to cells without treatment. (**D**) Effects of miR-200b inhibition on CDDP sensitivity in T24 cells. Cells were transfected with a miR-200b inhibitor or negative control (NC) and then treated with the indicated concentrations of CDDP. (**E**) Anti-tumor effects of the indicated concentrations of CDDP on EJ138 and EJ138RC cells. (**F**) qRT-PCR analysis of miR-200b in EJ138 and EJ138RC cells. Shown are means of 6 (A, C–E) or 3 (B, F) replications; error bars represent SDs. ^*^P < 0.05, ^**^P < 0.01.

We first established a CDDP-resistant BCa cell line by continuously exposing T24 cells to CDDP (T24RC), and confirmed the drug resistance in cell proliferation assays (Figure 1A). We next compared the miRNA expression profiles between T24RC and T24 cells using miRNA arrays, and found that 51 miRNAs were significantly downregulated in the resistant cells (>10-fold; Supplementary Table 1). In particular, miR-200 family members (miR-200b, miR-200a and miR-429) were at the top of the list, and subsequent qRT-PCR confirmed the decreased expression of miR-200b in T24RC (Table 1, Figure 1B). We next transfected T24RC cells with a miR-200a or miR-200b mimic and found that miR-200a suppressed cell proliferation but did not affect CDDP sensitivity (Supplementary Figure 1). On the other hand, exogenous expression of miR-200b in T24RC cells not only suppressed cell proliferation, it also partially restored the CDDP sensitivity (Figure 1C, Supplementary Figure 1). Conversely, inhibition of miR-200b diminished CDDP sensitivity in the parental cells (Figure 1D).

**Table 1:** Expression of top 10 miRNAs downregulated in T24RC as compared to T24 cells

	T24	T24RC	
miRNA name	miRNA/RNU6B	miRNA/RNU6B	Fold change
hsa-miR-200b-3p	4.31 × 10^–4^	1.58 × 10^–7^	3.67 × 10^–4^
hsa-miR-200a-3p	3.30 × 10^–4^	1.58 × 10^–7^	4.80 × 10^–4^
hsa-miR-505-3p	3.38 × 10^–5^	1.58 × 10^–7^	4.68 × 10^–3^
hsa-miR-9-5p	3.07 × 10^–5^	1.58 × 10^–7^	5.15 × 10^–3^
hsa-miR-429	9.05 × 10^–5^	5.52 × 10^–7^	6.11 × 10^–3^
hsa-miR-135b-3p	1.66 × 10^–5^	1.13 × 10^–7^	6.80 × 10^–3^
hsa-miR-150-5p	1.74 × 10^–5^	1.58 × 10^–7^	9.10 × 10^–3^
hsa-miR-192-3p	1.02 × 10^–5^	1.13 × 10^–7^	1.10 × 10^–2^
hsa-miR-34b-3p	1.41 × 10^–4^	1.58 × 10^–6^	1.12 × 10^–2^
hsa-miR-224-5p	1.19 × 10^–5^	1.58 × 10^–7^	1.33 × 10^–2^

To validate the results in other BCa cells, we conducted a qRT-PCR analysis in a series of BCa cell lines and detected abundant expression of miR-200b in EJ138 and HT1376 cells (Supplementary Figure 2). We then established a CDDP-resistant EJ138 line (EJ138RC, Figure 1E) and found that, as in T24RC cells, miR-200b expression was also reduced in EJ138RC cells (Figure 1F). Moreover, inhibition of miR-200b in EJ138 and HT1376 cells diminished their CDDP sensitivity (Supplementary Figure 2).

### miR-200b is epigenetically downregulated in CDDP-resistant BCa cells

**Figure 2:**
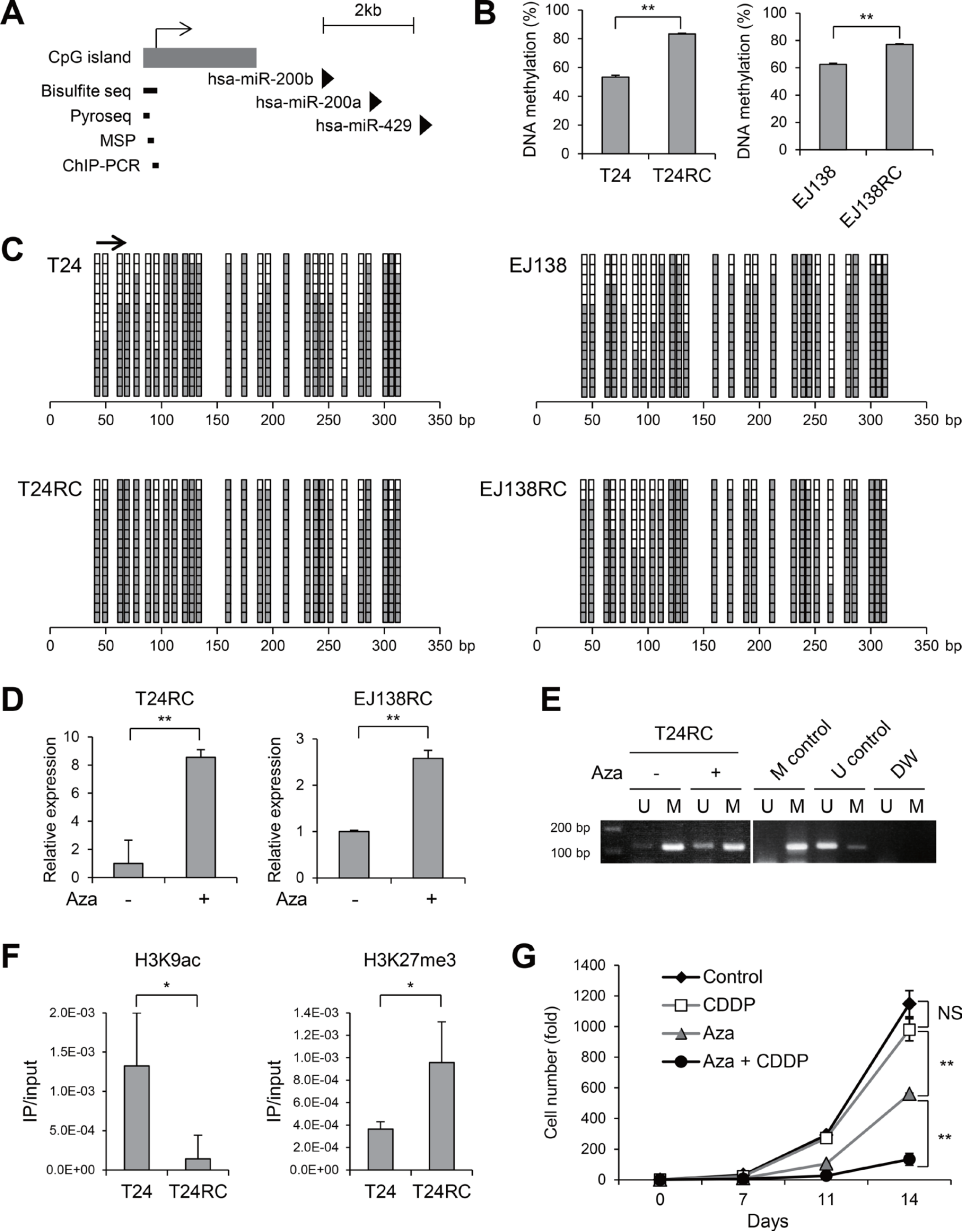
Epigenetic silencing of miR-200b in CDDP-resistant BCa cells (**A**) Schematic representation of the miR-200b, -200a and -429 genomic loci. Location of the CpG island and regions analyzed using the indicated methods are also shown. (**B**) Results of bisulfite pyrosequencing in the indicated BCa cells. (**C**) Results of bisulfite sequencing in the indicated cells. The region analyzed with bisulfite pyrosequencing is indicated by an arrow. (**D**) qRT-PCR analysis of miR-200b in CDDP-resistant BCa cells treated with or without 5-aza-dC (Aza). (**E**) MSP analysis in T24RC cells treated with or without 5-aza-dC. Bands in the “M” lanes are PCR products obtained with methylation-specific primers; those in the “U” lanes are products obtained with unmethylated-specific primers. *In vitro* methylated DNA and DNMT1/DNMT3B double knockout HCT116 cells serve as methylated and unmethylated controls. (**F**) ChIP-PCR analysis in the indicated cells. Levels of H3K9ac and H3K27me3 are shown. (**G**) Proliferation of T24RC cells treated with or without 5-aza-dC (Aza) and/or CDDP. Numbers of cells with the indicated treatments are shown relative to the numbers on day 0 (5 × 10^3^ cells). Shown in B, D, F and G are means of 3 replications; error bars represent SDs. NS, not significant; ^*^P < 0.05, ^**^P < 0.01.

Earlier studies showed that miR-200b is silenced in association with hypermethylation of its upstream CpG island in various malignancies, including breast, colon and bladder cancer [13, 14]. We therefore assessed the methylation status of the CpG island upstream of miR-200b in BCa cells (Figure 2A). Quantitative bisulfite pyrosequencing analysis revealed that, although both T24 and EJ138 cells exhibited substantial levels of methylation, even greater methylation was present in the resistant cells (Figure 2B). Bisulfite sequencing analysis also confirmed the increased methylation in CDDP-resistant cells (Figure 2C). Treatment with the DNA demethylating agent 5-aza-dC upregulated miR-200b expression in both T24RC and EJ138EC cells (Figure 2D). The presence of unmethylated DNA in T24RC cells treated with 5-aza-dC was confirmed using methylation-specific PCR (MSP; Figure 2E). To clarify the role of histone modifications in the repression of miR-200b in CDDP-resistant cells, we assessed acetylation of histone H3 lysine 9 (H3K9ac), a mark of active transcription, and trimethylation of H3K27 (H3K27me3), a repressive mark, in the CpG island region. Chromatin immunoprecipitation (ChIP)-PCR revealed lower levels of H3K9ac and higher levels of H3K27me3 in T24RC cells than in the parental cells (Figure 2F). Moreover, the combination of low-dose 5-aza-dC + CDDP strongly suppressed T24RC cell proliferation (Figure 2G). These results suggest miR-200b is epigenetically silenced in CDDP-resistant BCa cells and that this epigenetic alteration could be a therapeutic target to overcome drug resistance.

### Effects of miR-200b on gene expression profiles in CDDP-resistant BCa cells

**Figure 3:**
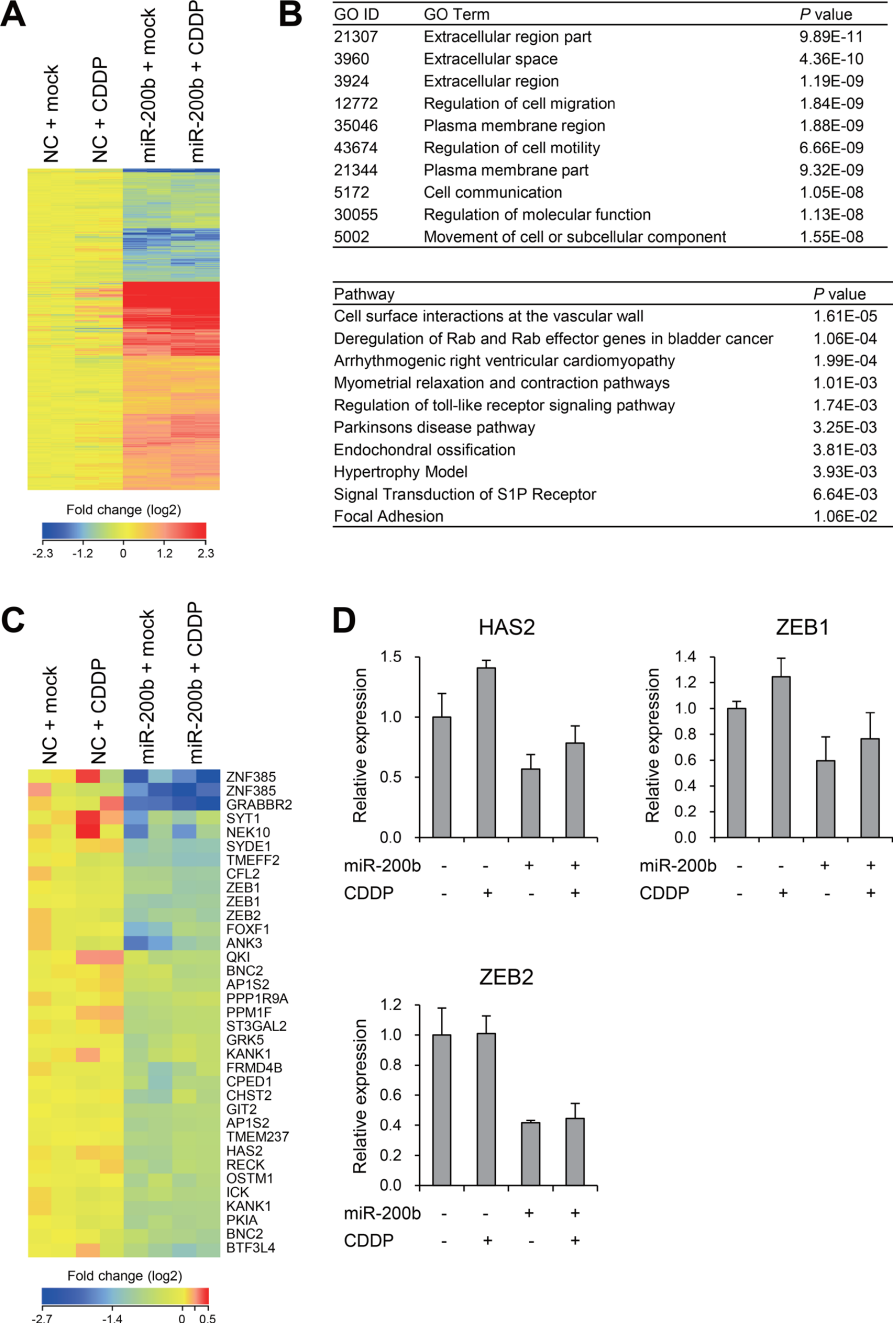
Effects of miR-200b on gene expression profiles in CDDP-resistant BCa cells (**A**) Heat map showing expression of 733 probe sets (595 genes) identified through microarray analysis of T24RC cells transfected with a miR-200b mimic or negative control (NC) and then treated with or without CDDP. (**B**) Results of gene ontology (GO, upper) and pathway (lower) analyses of the 595 selected genes. (**C**) Heat map showing expression of miR-200b target genes predicted by TargetScan. (**D**) qRT-PCR analysis of the indicated genes in T24RC cells with or without miR-200b and/or CDDP. Shown are means of 3 replications; error bars represent SDs.

To further explore the molecular mechanism by which miR-200b improves CDDP sensitivity, we carried out gene expression microarray analysis in T24RC cells transfected with a miR-200b mimic or a negative control and/or treated with or without CDDP. To assess the effects of miR-200b on gene expression profiles, we identified 733 probe sets (595 unique genes) differentially expressed between cells transfected with a negative control or miR-200b mimic (>1.5-fold, *P* < 0.05; Figure 3A, Supplementary Table 2). Gene ontology analysis revealed that genes associated with “extracellular space/region” and “regulation of cell migration/motility” were enriched among the selected genes, while pathway analysis suggested genes associated with “deregulation of Rab and Rab effector genes in bladder cancer” were enriched among the selected genes (Figure 3B). We then compared our microarray results with the list of miR-200b-target genes predicted by TargetScan and found that expression of 30 predicted target genes was decreased by miR-200b in T24RC cells (Figure 3C). In addition, RT-PCR analysis validated the expression of representative genes (HAS2, ZEB1 and ZEB2) reportedly associated with chemoresistance [15–17].

### miR-200b and CDDP activate genes associated with chemosensitivity and apoptosis

**Figure 4:**
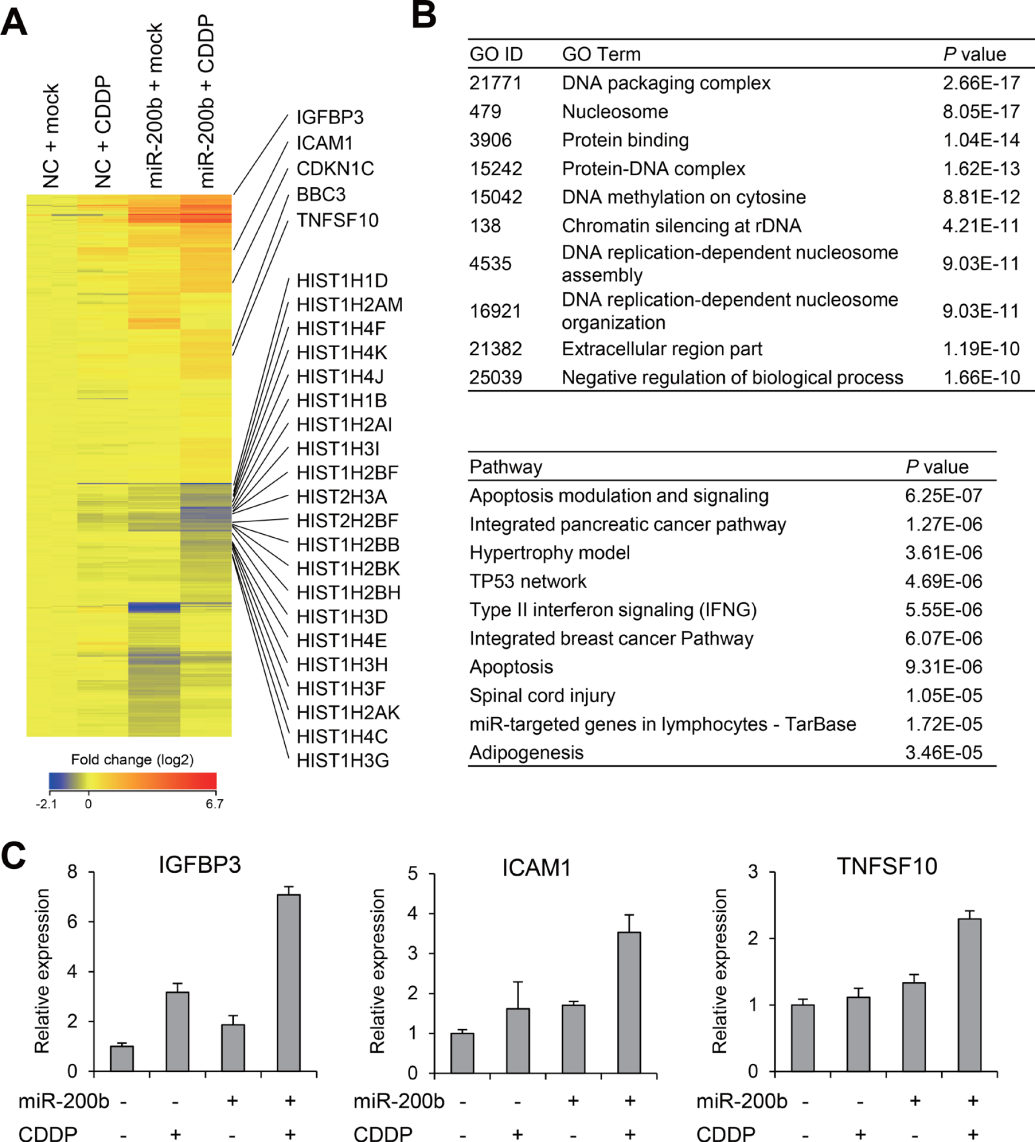
Effects of miR-200b and CDDP on gene expression profiles in CDDP-resistant BCa cells (**A**) Heat map showing expression of 551 probe sets (509 genes) identified through microarray analysis of T24RC cells transfected with a miR-200b mimic or negative control (NC) and then treated with or without CDDP. (**B**) Results of gene ontology (GO, upper) and pathway analyses (lower) of the selected 509 genes. (**C**) qRT-PCR analysis of the indicated genes in T24RC cells with or without miR-200b and/or CDDP. Shown are means of 3 replications; error bars represent SDs.

We next assessed the effects of CDDP/miR-200b and found that, by itself, CDDP had only limited effects on gene expression, whereas miR-200b induced significant changes in the gene expression profile of T24RC cells (Supplementary Figure 3). We then selected genes that were differentially expressed between T24RC cells treated with miR-200b alone and those treated with miR-200b + CDDP (*P* < 0.05), and identified a series of 551 probe sets corresponding to 509 unique genes (Figure 4A, Supplementary Table 3). Gene ontology analysis revealed that genes associated with “DNA packaging complex” and “nucleosome” were enriched among 509 selected genes (Figure 4B). Consistent with this result, a number of genes encoding histones were downregulated by miR-200b + CDDP, perhaps reflecting inhibition of DNA replication and the cell cycle (Figure 4A). Pathway analysis suggested that genes associated with “apoptosis modulation and signaling” were also enriched among the selected genes (Figure 4B). Consistent with this finding, apoptosis-related genes, including TNFSF10 (also known as tumor necrosis factor-related apoptosis-inducing ligand, TRAIL) and BBC3 (also known as PUMA), were upregulated by miR-200b + CDDP (Figure 4A). We also found that IGFBP3 and ICAM1, which are reportedly associated with CDDP resistance, were synergistically upregulated by miR-200b + CDDP [18–20] (Figure 4A). These microarray results were validated for selected genes using qRT-PCR (Figure 4C). Collectively then, these results suggest miR-200b sensitizes BCa cells to CDDP by inducing multiple genes involved in determining chemosensitivity and cytotoxicity.

### Expression and methylation of miR-200b is associated with BCa prognosis

**Figure 5:**
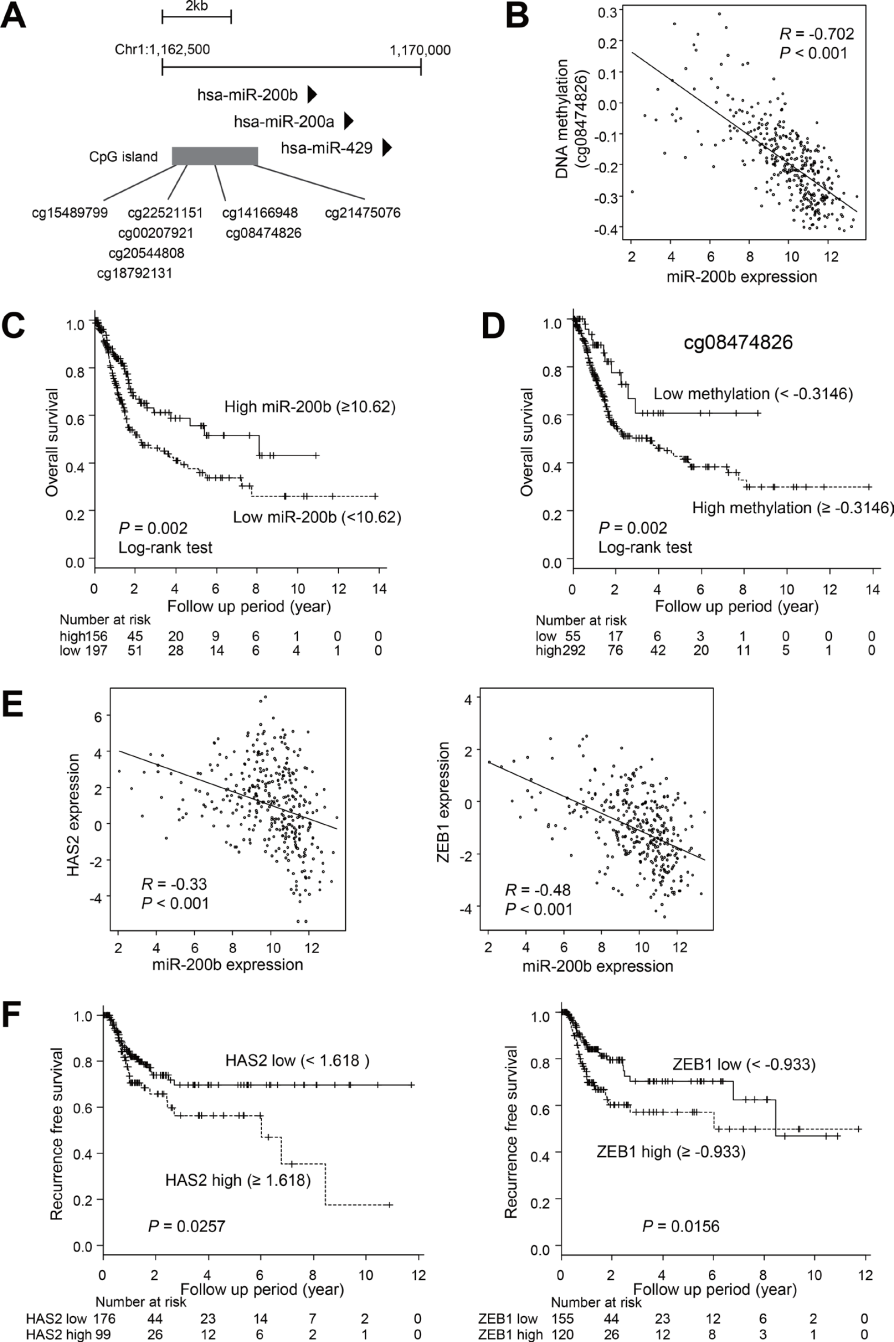
Analysis of the expression and DNA methylation of miR-200b in clinical BCa tumors using TCGA data sets (**A**) Locations of Infinium BeadChip probes in the CpG island of miR-200b. Genomic locations are based on Human GRCh38/hg38. (**B**) Correlation between levels of miR-200b expression and DNA methylation of the indicated probe in primary BCa (*n* = 345). The Pearson's correlation coefficient (R) is also shown. (**C**) Kaplan–Meier curve showing the effect of miR-200b expression on overall survival of BCa patients (*n* = 353). (**D**) Kaplan–Meier curve showing the effect of DNA methylation of the indicated probe on overall survival of BCa patients (*n* = 347). (**E**) Correlation between levels of miR-200b expression and expression of HAS2 (left) or ZEB1 (right) in primary BCa (*n* = 345). (**F**) Kaplan–Meier curves showing the effects of HAS2 (left) and ZEB1 (right) expression on recurrence-free survival among patients with stage 2 or higher BCa (*n* = 275).

We next evaluated the clinical relevance of our findings. Using data sets for primary BCa from The Cancer Genome Atlas (TCGA), we compared expression of miR-200b and DNA methylation of its upstream CpG island [21]. Because chemotherapy is not commonly administered to patients with NMIBC, we selected the data from patients with stage II or higher tumors. We found that methylation levels at multiple CpG sites in the CpG island correlated inversely with the levels of miR-200b expression in BCa (Figure 5A, 5B, Supplementary Figure 4). Kaplan–Meier curve analysis revealed that lower levels of miR-200b expression and higher levels of DNA methylation were associated with poor overall survival (Figure 5C, 5D, Supplementary Figure 5). Logistic regression analysis revealed that higher levels of miR-200b expression (≥10.62) were associated with better overall survival (HR, 0.57; 95% CI, 0.4–0.82; *P* < 0.01). Similarly, lower methylation levels (cg0847826 < –0.3146) were associated with better overall survival (HR, 0.47; 95% CI, 0.25–0.88; *P* < 0.01). These results suggest that miR-200b is frequently silenced in association with DNA methylation in primary BCa tumors, and that epigenetic silencing of miR-200b is associated with a poorer outcome of BCa.

Using the TCGA data sets, we also confirmed that levels of miR-200b expression are inversely associated with those of HAS2, ZEB1 and ZEB2 (Figure 5E, Supplementary Figure 6). Moreover, higher HAS2 and ZEB1 expression was associated with poorer recurrence-free survival among patients with stage 2 or higher BCa (Figure 5F), whereas there was no significant association between ZEB2 expression and clinical outcome (Supplementary Figure 6).

## DISCUSSION

In the present study, we found that expression of a number of miRNAs is altered after acquisition of CDDP resistance by BCa cells. We detected significant downregulation of miR-200 family members, including miR-200b, -200a and -429 in CDDP-resistant cells. The simultaneous reduction of these miRNAs is thought to be associated with increased DNA methylation of their upstream CpG islands. But among these three molecules, it was downregulation of miR-200b that was associated with CDDP resistance in BCa cells. The miR-200 family suppresses epithelial-to-mesenchymal transition (EMT) by targeting E-cadherin repressors ZEB1 and ZEB2 [22, 23]. Loss of the miR-200 family promotes EMT and invasive and aggressive cancer phenotypes [14, 24]. miR-200 family genes are encoded in two polycistronic units, miR-200b/200a/429 on chromosome 1 and miR-200c/141 on chromosome 12 [14]. CpG islands are located in the upstream of both units, and they are targets of hypermethylation in various cancers, including BCa [14]. Epigenetic silencing of the miR-200 family is seen in MIBC tumors and undifferentiated BCa cell lines [13].

Several studies have implicated the miR-200 family in chemoresistance. For instance, the CDDP-resistant variant of MCF7 breast cancer cells expresses lower levels of miR-200b and miR-200c than the parental cells [25]. Similarly, miR-200b expression is decreased in CDDP-resistant tongue cancer cells, and ectopic expression of miR-200b sensitizes the cells to chemotherapy [26]. Subsequent studies also showed that increasing miR-200b expression leads to improved chemosensitivity in lung and esophageal cancer cells [27, 28]. More recently, a study using a mouse model to track EMT during metastasis of breast tumors showed that miR-200 not only inhibits EMT but also reduces chemoresistance to cyclophosphamide [29]. By contrast, other studies showed that miR-200b/200c/429 could induce chemoresistance in endometrial cancer and Ehrlich ascites tumor cells, suggesting the miR-200 family may exert opposite effects in different tumor types [30, 31]. To our knowledge, this is the first study to show an association between miR-200b downregulation and CDDP resistance in BCa.

Our microarray analysis revealed that ectopic expression of miR-200b significantly altered expression of a number of genes, including well-characterized miR-200-targets in CDDP-resistant BCa cells. Notably, ZEB1 and ZEB2 are implicated in chemoresistance in a variety cancer types. For instance, ZEB1 induces CDDP resistance in tongue cancer cells by transcriptionally activating carbonic anhydrase 9 (CA9) [16]. Another study showed that miR-128 sensitizes prostate cancer cells to CDDP by suppressing ZEB1 [32]. Similarly, miR-200c, miR-203 and miR-218 increase CDDP-sensitivity via targeting ZEB2 in gastric, nasopharyngeal and lung cancers [17, 33, 34]. Hyaluronan (HA) is a major ligand of CD44, and the HA-CD44 signal promotes cell adhesion, migration and proliferation [35]. The oncogenic HA-CD44 signal is also involved in chemoresistance, and hyaluronan synthase 2 (HAS2) reportedly induces CD44-dependent CDDP resistance in oral cancer [15]. Treatment with carboplatin significantly increases expression of HAS2 and HAS3, and the resultant increase of HA secretion may contribute to chemoresistance in ovarian cancer [36]. These results suggest suppressing miR-200b may induce CDDP resistance by upregulating its target genes in BCa cells.

We also found that miR-200b and CDDP act synergistically to upregulate multiple genes associated with chemosensitivity or cytotoxicity in CDDP-resistant BCa cells. IGFBP3 is a key regulator of insulin growth factor (IGF) signaling. IGF1 binds to IGFBP3 with higher affinity than for its specific receptor (IGF1R), which disrupts interaction between IGF1 and IGF1R and suppresses the mitogenic and anti-apoptotic actions by IGF1 [18]. An earlier study showed that IGFBP3 is silenced by CpG island hypermethylation in CDDP-resistant NSCLC, and that IGFBP3 knockdown diminishes CDDP sensitivity in NSCLC cells [18]. A subsequent study demonstrated that methylation of IGFBP3 mediates CDDP resistance by activating the IGF1R/Akt pathway [19]. Loss of IGFBP3 also mediates acquired resistance to EGFR tyrosine kinase inhibitors [37], and upregulation of IGFBP3 is reportedly associated with increased chemosensitivity of esophageal cancer cells to nimotuzumab (anti-EGFR monoclonal antibody) with CDDP [38].

A recent study searched for genes differentially expressed and methylated in CDDP-resistant NSCLC cells and their parental cells [20]. Among the genes identified, ICAM1 was confirmed to be silenced by DNA methylation in CDDP-resistant cells. Although functional association between ICAM1 and CDDP sensitivity has not been fully characterized, an earlier study showed that the combination of CDDP + 5-fluorouracil synergistically induced ICAM1 expression in cancer cells, which may facilitate recognition of cancer cells by T lymphocytes [39]. CDDP also induces ICAM1 expression in endothelial cells via a NF-κB dependent pathway, which could lead to CDDP-induced vascular toxicity [40]. In addition, it was recently reported that upregulation of ICAM1 suppresses growth of ovarian cancer cells in the absence of immune cells [41]. However, this study also showed that induction of ICAM1 leads to reduced CDDP sensitivity in ovarian cancer cells. Further study will be necessary to clarify the role of ICAM1 in BCa.

TNFSF10 (tumor necrosis factor ligand super family member 10), also known as TRAIL, is a member of the TNF superfamily that induces apoptosis in various cancers by activating death receptors [42]. Although TRAIL is not reportedly associated with CDDP sensitivity, multiple lines of evidence show that CDDP enhances susceptibility to TRAIL-mediated apoptosis in various malignancies, including esophageal, lung and bladder cancers [43–46]. More recently, a study showed that CDDP-resistant cancer cells are sensitive to TRAIL-mediated apoptosis [47]. This suggests induction of TRAIL by miR-200b and CDDP may lead to increased cytotoxicity in BCa cells.

Our study has several limitations. Although our data suggest miR-200b is epigenetically silenced in CDDP-resistant BCa cells, the CpG island is already highly methylated in the parental cells, and 5-aza-dC treatment induces only moderate re-expression of miR-200b. These findings together with our ChIP-PCR results suggest both DNA methylation and histone modification are involved in suppressing miR-200b. Consequently, the clinical usefulness of DNA methylation as a predictive marker of CDDP resistance may be limited. In addition, due to a lack of clinical information, we were unable analyze the association between miR-200b expression and clinical outcomes in BCa patients treated with CDDP. Nonetheless, the combination of low dose 5-aza-dC and CDDP strongly suppressed proliferation of CDDP-resistant BCa cells. This suggests multiple factors involved in chemosensitivity may be epigenetically silenced in CDDP-resistant BCa, and that epigenetic drug treatment could be a therapeutic option for such tumors. Further study is warranted to clarify the clinical implications and usefulness of miR-200b in BCa.

In summary, our data suggest that downregulation of miR-200b is associated with CDDP resistance in BCa, and that restoration of miR-200b or treatment with a DNA demethylating agent could be an effective therapeutic approach to overcoming chemoresistance. Moreover, epigenetic alteration of miR-200b may be a predictive marker of BCa chemoresistance and useful for identifying patients who will benefit from the neoadjuvant chemotherapy.

## MATERIALS AND METHODS

### Cell lines

The T24, UMUC3 and EJ138 BCa cell lines were obtained from The European Collection of Authenticated Cell Cultures (ECACC). TCCSUP, J82 and HT1376 cells were obtained from the American Type Culture Collection (ATCC). T24 cells were cultured in McCoy's 5A medium (Thermo Fisher Scientific, Waltham, MA, USA) supplemented with 10% fetal bovine serum (FBS). The other BCa cell lines were cultured in Eagle's minimal essential medium (EMEM; Wako Pure Chemical Industries, Osaka, Japan) with 10% FBS. To establish CDDP-resistant BCa cells (T24RC and EJ138RC), T24 and EJ138 cells were continuously exposed to serially elevated concentrations (up to 3 μg/ml) of CDDP for >6 months. CDDP-resistant BCa cells were maintained in culture medium with 3 μg/ml CDDP. To restore epigenetically silenced genes, cells were treated with 1 μM 5-aza-2’-deoxicytidine (5-aza-dC; Wako, Tokyo, Japan) for 72 h. Total RNA was extracted using TRI Reagent (COSMO BIO, Tokyo, Japan), and genomic DNA was extracted using the standard phenol-chloroform procedure.

### miRNA expression

Expression of a series of 754 miRNAs was examined using a TaqMan MicroRNA Array v3.0, which includes qRT-PCR assays for 754 human miRNAs (Thermo Fisher Scientific, Waltham, MA, USA). The PCR was run in a 7900HT Fast Real-Time PCR System (Thermo Fisher Scientific), and SDS 2.2.2 software (Thermo Fisher Scientific) was used for comparative delta Ct analysis. U6 snRNA (RNU6B) was used as an endogenous control. Expression of miR-200b and RNU6B was assessed using TaqMan MicroRNA Assays (Assay ID, 00251 and 001093, Thermo Fisher Scientific) and a 7500 Fast Real-Time PCR System (Thermo Fisher Scientific).

### Transfection of miRNA mimics or inhibitors

For ectopic expression of miRNAs, BCa cells plated at 5 × 10^5^ cells/well in 6-well dishes were transfected with 25 pmol of mirVana miRNA mimic (hsa-miR-200a-3p, hsa-miR-200b-3p, Thermo Fisher Scientific) or mirVana miRNA mimic Negative Control #1 (Thermo Fisher Scientific) using Lipofectamine RNAiMAX (Thermo Fisher Scientific) according to manufacturer's instructions. For suppression of miRNAs, cells were transfected with 25 pmol of mirVana miRNA inhibitor (hsa-miR-200b-3p, hsa-miR-661, hsa-miR-21-5p, Thermo Fisher Scientific) or mirVana miRNA inhibitor Negative Control #1 (Thermo Fisher Scientific) using Lipofectamine RNAiMAX.

### Drug treatment and cell proliferation assay

To assess drug sensitivity, BCa cells (1 × 10^4^ cells/well in 24-well plates) were treated with or without CDDP (0.2 to 3.2 μg/ml) for 72 h, after which cell numbers were counted using a Countess II Automated Cell Counter (Thermo Fisher Scientific). To assess the involvement of miRNAs in the drug sensitivity, BCa cells were transfected with a miRNA mimic or inhibitor as described above and incubated for 48 h. Cells were then trypsinized, counted, placed in 24-well plates (1 × 10^4^ cells/well), and incubated for additional 24 h. Thereafter, cells were treated with or without CDDP for 72 h, after which cell numbers were counted. For combined treatment with 5-aza-dC + CDDP, BCa cells (5 × 10^5^ cells in a 10-cm dish) were treated with or without 100 nM 5-aza-dC for 48 h, after which the medium was replaced and the cells were treated with or without 3.0 μg/ml CDDP for additional 72 h. After removing the drugs, 5 × 10^3^ cells were placed in a 10-cm dish, and cell numbers were counted 7, 11 and 14 days after the start of drug treatment.

### DNA methylation analysis

Genomic DNA (1 μg) was modified with sodium bisulfite using an EpiTect Bisulfite kit (Qiagen, Hilden, Germany). Bisulfite sequencing, bisulfite pyrosequencing and methylation-specific PCR (MSP) were conducted as described previously [48]. *In vitro* methylated DNA and DNMT1/DNMT3B double knockout HCT116 cells served as methylated and unmethylated controls [48]. The pyrosequencing reaction was performed using a PSQ96 system with a PyroGold reagent Kit (Qiagen), and the results were analyzed using Q-CpG software (Qiagen). For bisulfite sequencing of the CpG island of miR-200b, a region including 27 CpG sites was PCR amplified and cloned into pCR2.1-TOPO vector (Life Technologies), after which 14 or 15 clones from each sample were sequenced using an ABI3130x automated sequencer (Thermo Fisher Scientific). Primer sequences and PCR product sizes are listed in Supplementary Table 4.

### Chromatin immunoprecipitation (ChIP)-PCR

ChIP was carried out using anti-acetyl-histone H3 lysine 9 (#07-352, Millipore, Billerica, Massachusetts, USA) and anti-H3K27me3 (#9733, Cell Signaling Technology, Danvers, MA, USA) antibodies as described previously [49]. Input DNA and the immunoprecipitates were subjected to quantitative PCR analysis using PowerUp SYBR Green PCR Master Mix (Life Technologies). Primer sequences and PCR product sizes are listed in Supplementary Table 4.

### Gene expression microarray analysis

T24RC cells were transfected with a miR-200b mimic or negative control and then treated with or without 1.6 μg/ml CDDP as described above. One-color microarray-based gene expression analysis was then carried out according to manufacturer's instructions (Agilent Technologies, Santa Clara, CA, USA). Briefly, 100 ng of total RNA were amplified and labeled using a Low-input Quick Amp Labelling kit One-color (Agilent Technologies), after which the synthesized cRNA was hybridized to a SurePrint G3 Human GE microarray v2 (G4851; Agilent Technologies). Each sample was hybridized to 2 arrays, and the microarray data were analyzed using GeneSpring GX version 13 (Agilent Technologies). miR-200b target genes were predicted by TargetScan. (www.targetscan.org). The Gene Expression Omnibus accession number for the microarray data is GSE103250.

### Quantitative reverse transcription PCR

Single-stranded cDNA was prepared using SuperScript III reverse transcriptase (Thermo Fisher Scientific). Quantitative reverse transcription PCR (qRT-PCR) for IGFBP3, ICAM1 and ACTB was carried out using TaqMan Gene Expression Assays (Assay ID, Hs00426287_m1, Hs00164932_m1 and Hs01060665_g1, Thermo Fisher Scientific). ACTB (β-actin) was used as an endogenous control. qRT-PCR for HAS2, ZEB1, ZEB2 and TNFSF10 was performed using PowerUp SYBR Master Mix (Thermo Fisher Scientific). The PCR was run in a 7500 Fast Real-Time PCR System (Thermo Fisher Scientific), and SDS ver. 1.4 (Thermo Fisher Scientific) was used for comparative delta Ct analysis. Primer sequences for TNFSF10 are listed in Supplementary Table 4.

### Statistical analysis

Comparisons of continuous variables were made using *t* tests or one-way ANOVA with post-hoc multiple comparisons (Tukey HSD test). Correlations were evaluated based on Pearson's correlation coefficient. Values of *P* < 0.05 (two-sided) were considered significant. Optimal cut off levels were determined through receiver operating characteristic (ROC) curve analysis. Differences in survival were compared using the Kaplan–Meier method with the Log-rank test. All data were analyzed using EZR ver. 1.35 (Saitama Medical Center, Jichi Medical University) [50].

## SUPPLEMENTARY MATERIALS FIGURES AND TABLES







## Abbreviations

BCa: bladder cancer
NMIBC: non-muscle invasive bladder cancer
MIBC: muscle invasive bladder cancer
CDDP: cisplatin
