# Supplementary material for: Epigenetic silencing of miR-200b is associated with cisplatin resistance in bladder cancer
Source: Oncotarget. 2018 May 11;9(36):24457–69. doi: 10.18632/oncotarget.25326 (PMC5966259; doi:10.18632/oncotarget.25326)
Supplement: Supplementary file 1 [file oncotarget-09-24457-s001.pdf]

Epigenetic silencing of miR-200b is associated with cisplatin resistance in bladder cancer

SUPPLEMENTARY MATERIALS

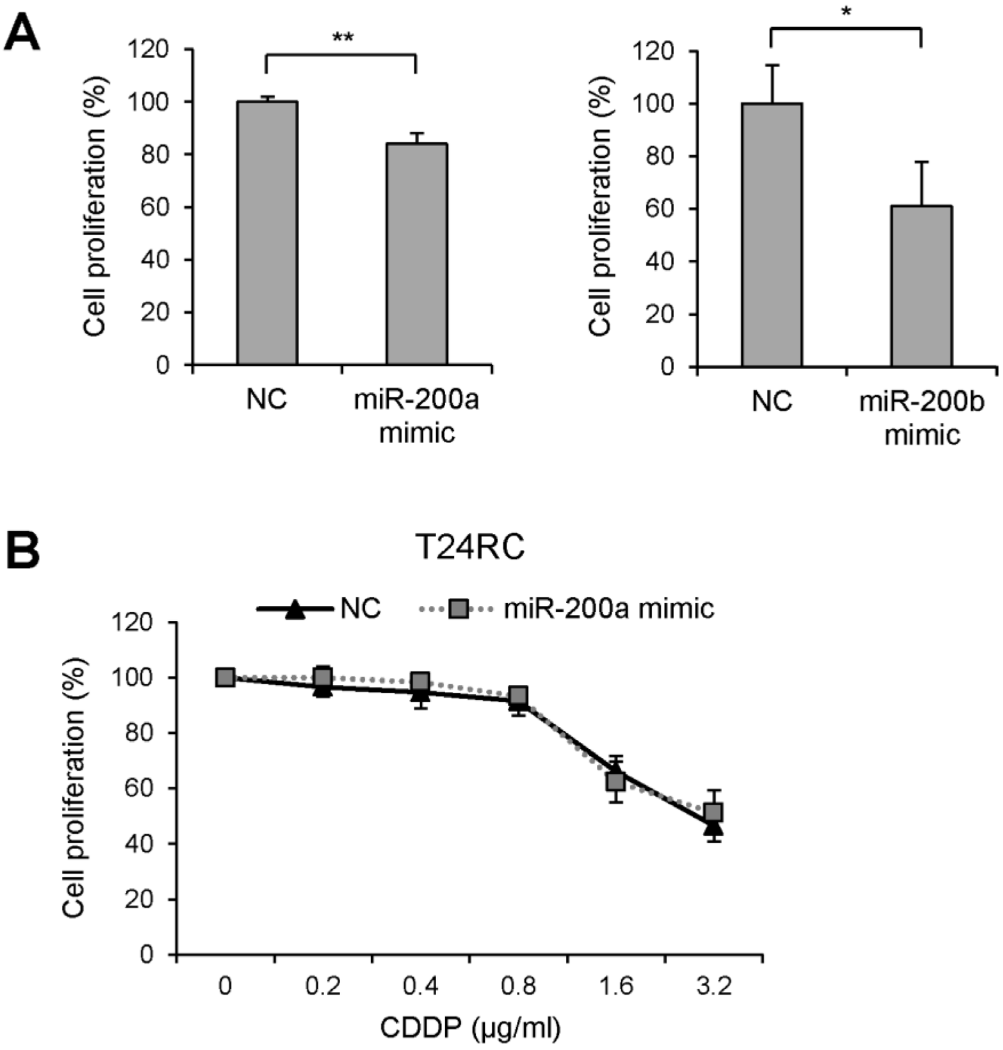

Supplementary Figure 1: Effects of miR-200a/b on proliferation and CDDP sensitivity in BCa cells. (**A**) Effects of miR-200a (left) and miR-200b (right) on proliferation of T24RC cells. Cells were transfected with a miRNA mimic or negative control (NC), and cell numbers were counted 96 h after transfection. (**B**) Effects of miR-200a on CDDP sensitivity in T24RC cells. Numbers of cells transfected with a miR-200a mimic or negative control (NC) and then treated with the indicated concentrations of CDDP are shown relative to the numbers without treatment. $^{*}P < 0.05$, $^{**}P < 0.01$.

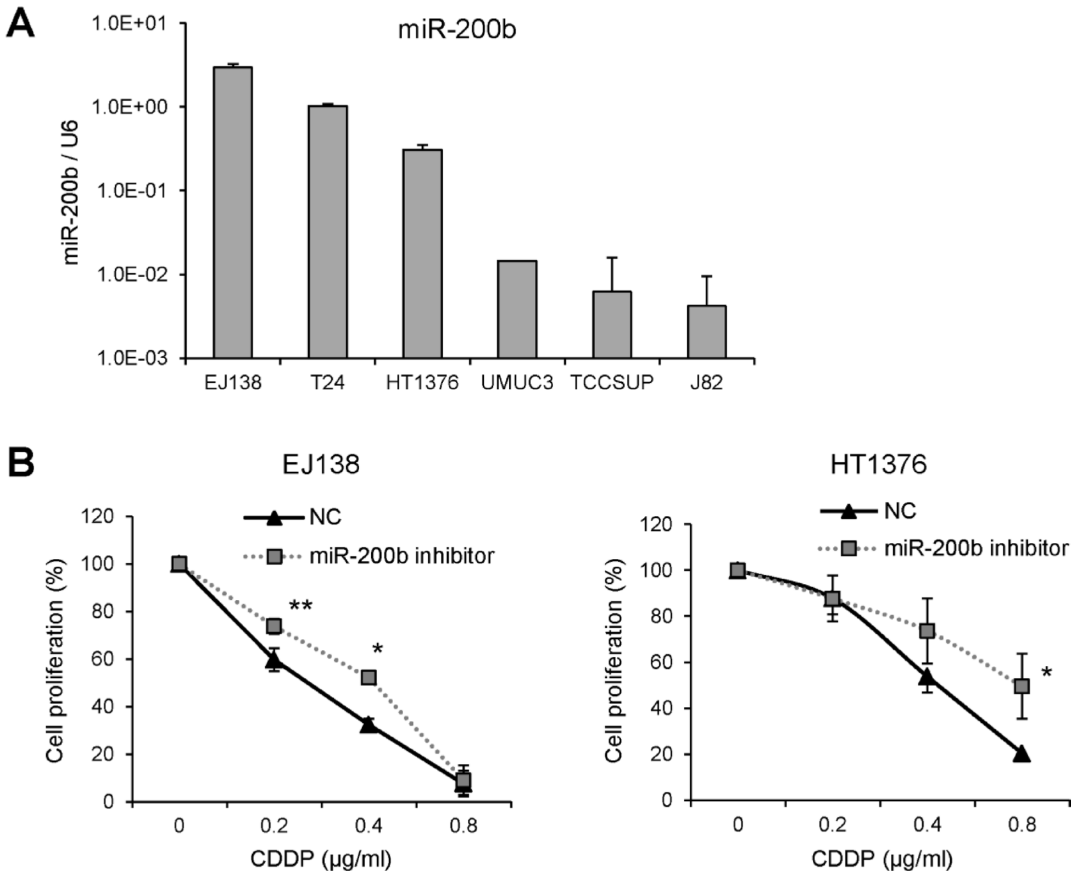

Supplementary Figure 2: Effects of miR-200b on CDDP sensitivity in BCa cells. (**A**) qRT-PCR analysis of miR-200b in the indicated BCa cell lines. (**B**) Effects of miR-200b inhibition on CDDP sensitivity in the indicated BCa cells. Cells were transfected with a miR-200b inhibitor or negative control (NC) and then treated with the indicated concentrations of CDDP. Shown are the means of 3 (A) or 6 (B) replicates. Error bars represent SDs. $^{*}P < 0.05$, $^{**}P < 0.01$.

A

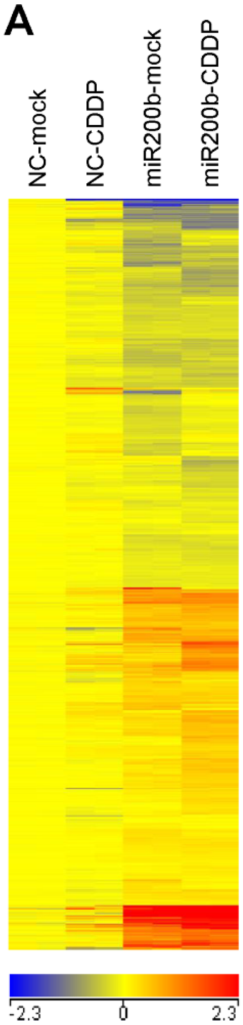

B

GO ID	GO Term	*P* value
21404	cytoplasmic part	5.32E-29
21385	intracellular part	3.70E-28
3974	intracellular	3.04E-27
4052	cytoplasm	1.83E-26
20254	membrane-bounded organelle	6.44E-25
4124	cytosol	2.01E-23
20253	organelle	3.18E-23
3906	protein binding	6.51E-22
30092	extracellular exosome	9.42E-22
38884	extracellular vesicle	9.42E-22

Pathway	*P* value
Hs_Histone_Modifications_WP2369_69927	9.70E-11
Hs_Apoptosis_Modulation_and_Signaling_WP1772_63162	1.81E-10
Hs_Apoptosis_WP254_72110	2.49E-10
Hs_Focal_Adhesion_WP306_71714	2.76E-10
Hs_Integrated_Breast_Cancer_Pathway_WP1984_72732	2.88E-10
Hs_Myometrial_Relaxation_and_Contraction_Pathways_WP289_72107	3.07E-10
Hs_miR-targeted_genes_in_epithelium_-_TarBase_WP2002_64823	3.60E-10
Hs_miR-targeted_genes_in_muscle_cell_-_TarBase_WP2005_69025	7.41E-10
Hs_miR-targeted_genes_in_lymphocytes_-_TarBase_WP2004_68896	7.66E-10
Hs_Integrated_Pancreatic_Cancer_Pathway_WP2377_71228	7.76E-10

Supplementary Figure 3: Effects of miR-200b and CDDP on gene expression profiles in CDDP-resistant BCa cells. (**A**) Heat map showing expression of 4241 probe sets (3559 unique genes) identified through microarray analysis in T24RC cells transfected with a miR-200b mimic or negative control (NC) and then treated with or without CDDP. Respective samples were analyzed in duplicate, and one-way ANOVA was performed to identify genes differentially expressed among the samples. (**B**) Results of gene ontology (GO, upper) and pathway analyses (lower) of the selected 3559 genes.

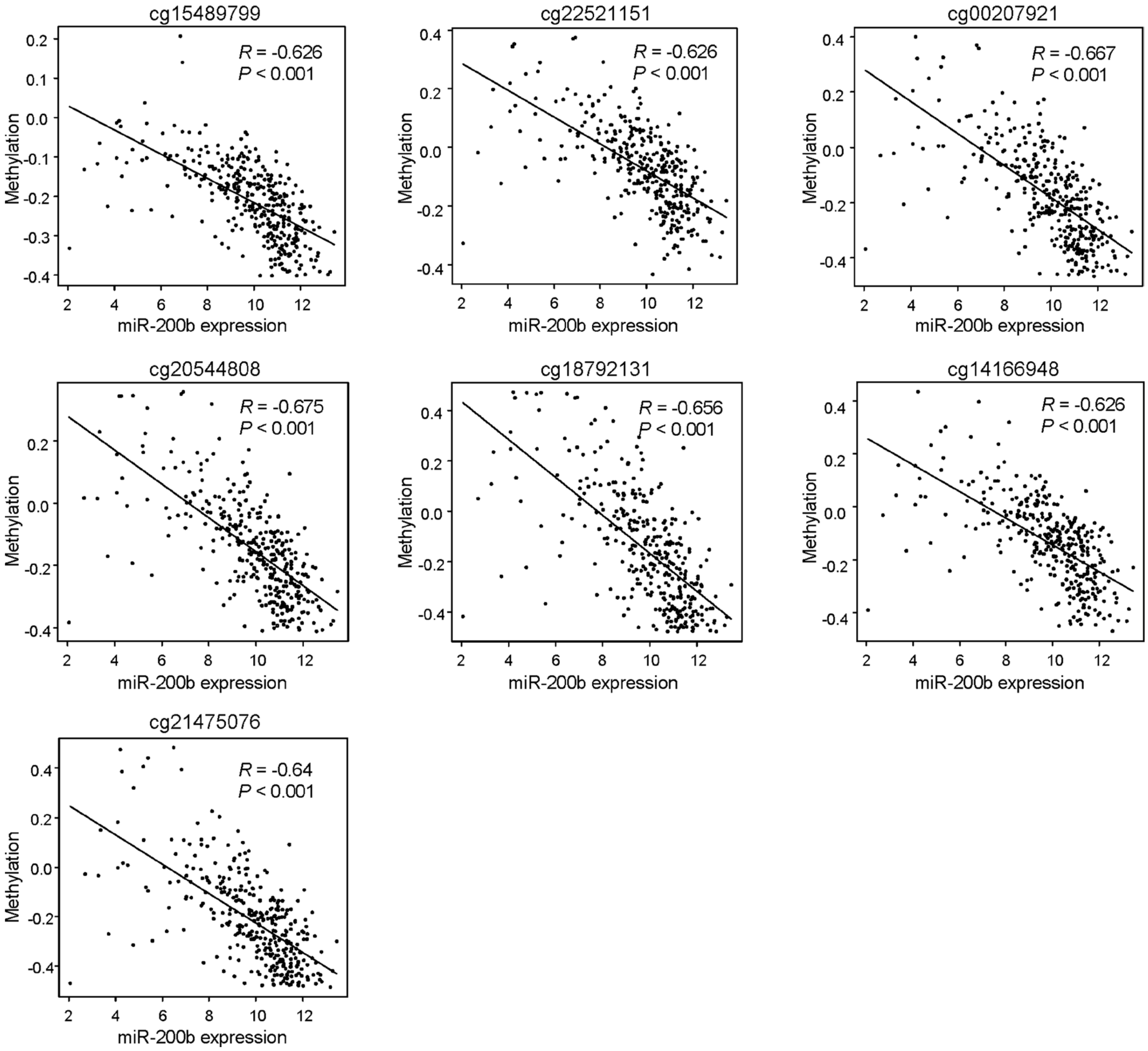

Supplementary Figure 4: Correlations between levels of miR-200b expression and DNA methylation of the indicated probes in TCGA data sets in primary BCa (*n* = 345). Pearson's correlation coefficients (*R*) are also shown.

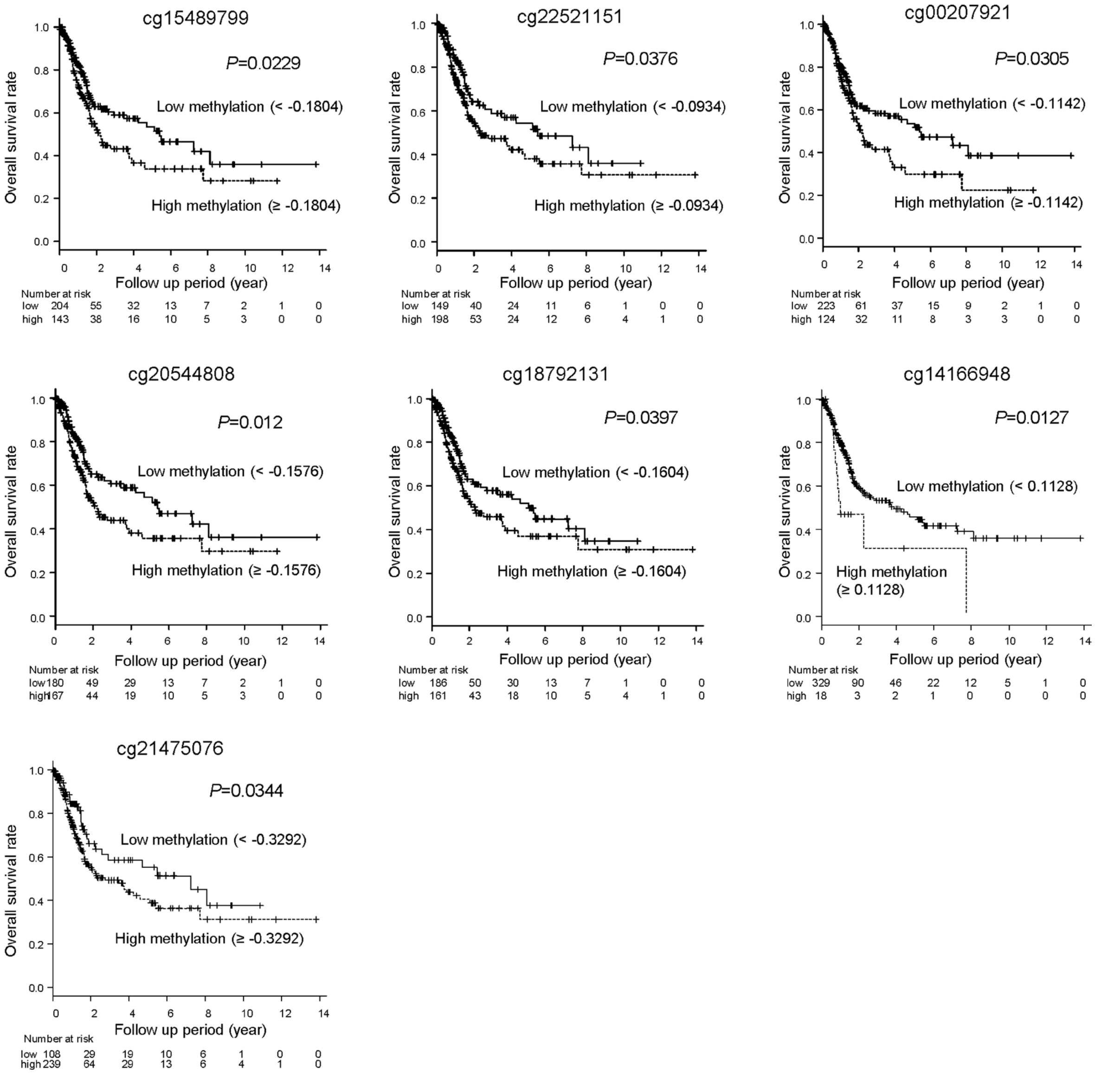

Supplementary Figure 5: Kaplan–Meier curves showing the effect of DNA methylation of the indicated probes in TCGA data sets on overall survival of BCa patients (*n* = 347).

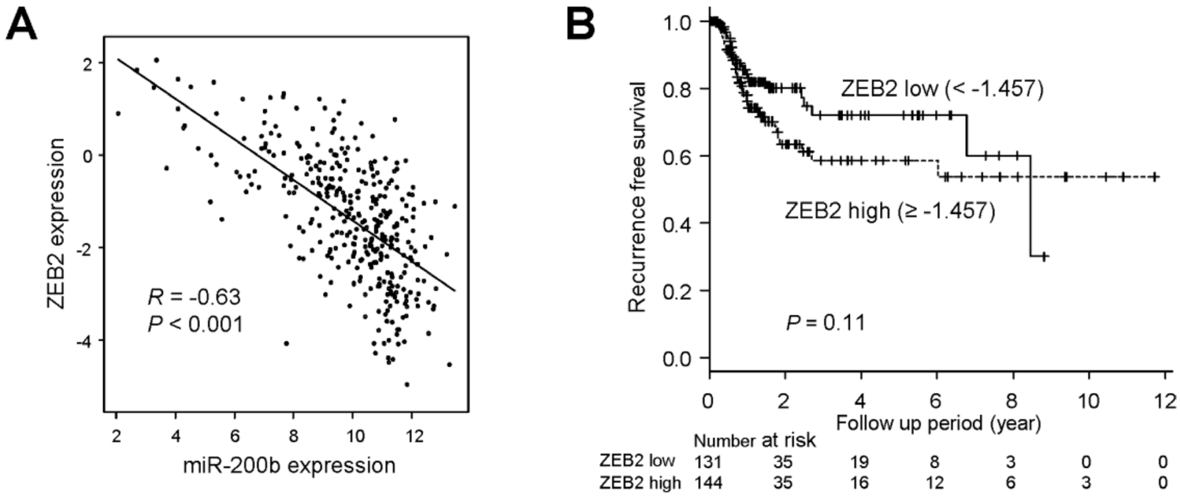

Supplementary Figure 6: Analysis of miR-200b and ZEB2 expression in clinical BCa tumors using TCGA data sets. (**A**) Correlation between levels of miR-200b and ZEB2 expression in primary BCa (n = 345). (**B**) Kaplan–Meier curves showing the effect of ZEB2 expression on recurrence-free survival among patient with stage 2 or higher BCa (n = 275).

Supplementary Table 1: Expression of miRNAs downregulated in T24RC as compared to T24 cells

	T24	T24RC	
miRNA name	miRNA/RNBU6	miRNA/RNBU6	Fold change
hsa-miR-200b-3p	4.31×10^{-4}	1.58×10^{-7}	3.67×10^{-4}
hsa-miR-200a-3p	3.30×10^{-4}	1.58×10^{-7}	4.80×10^{-4}
hsa-miR-505-3p	3.38×10^{-5}	1.58×10^{-7}	4.68×10^{-3}
hsa-miR-9-5p	3.07×10^{-5}	1.58×10^{-7}	5.15×10^{-3}
hsa-miR-429	9.05×10^{-5}	5.52×10^{-7}	6.11×10^{-3}
hsa-miR-135b-3p	1.66×10^{-5}	1.13×10^{-7}	6.80×10^{-3}
hsa-miR-150-5p	1.74×10^{-5}	1.58×10^{-7}	9.10×10^{-3}
hsa-miR-192-3p	1.02×10^{-5}	1.13×10^{-7}	1.10×10^{-2}
hsa-miR-34b-3p	1.41×10^{-4}	1.58×10^{-6}	1.12×10^{-2}
hsa-miR-224-5p	1.19×10^{-5}	1.58×10^{-7}	1.33×10^{-2}
hsa-miR-656-3p	7.53×10^{-6}	1.13×10^{-7}	1.50×10^{-2}
hsa-miR-576-3p	1.27×10^{-4}	1.96×10^{-6}	1.54×10^{-2}
hsa-miR-23a-3p	1.10×10^{-5}	1.58×10^{-7}	1.57×10^{-2}
hsa-miR-216b	9.84×10^{-6}	1.58×10^{-7}	1.61×10^{-2}
hsa-miR-34b-3p	6.95×10^{-6}	1.13×10^{-7}	1.62×10^{-2}
hsa-miR-614	6.79×10^{-6}	1.13×10^{-7}	1.66×10^{-2}
hsa-miR-539-5p	9.33×10^{-6}	1.58×10^{-7}	1.69×10^{-2}
hsa-miR-95	8.87×10^{-6}	1.58×10^{-7}	1.78×10^{-2}
hsa-miR-323-3p	1.78×10^{-5}	3.21×10^{-7}	1.80×10^{-2}
hsa-miR-203	8.75×10^{-6}	1.58×10^{-7}	1.81×10^{-2}
hsa-miR-193a-3p	8.33×10^{-6}	1.58×10^{-7}	1.90×10^{-2}
hsa-miR-516-3p	5.74×10^{-6}	1.13×10^{-7}	1.97×10^{-2}
hsa-miR-522-3p	5.66×10^{-5}	1.13×10^{-6}	2.00×10^{-2}
hsa-miR-1257	5.56×10^{-6}	1.13×10^{-7}	2.03×10^{-2}
hsa-miR-643	5.35×10^{-6}	1.13×10^{-7}	2.11×10^{-2}
hsa-miR-891a	7.14×10^{-6}	1.58×10^{-7}	2.22×10^{-2}
hsa-miR-193a-5p	7.03×10^{-6}	1.58×10^{-7}	2.25×10^{-2}
hsa-miR-564	4.78×10^{-6}	1.13×10^{-7}	2.36×10^{-2}
hsa-miR-517c-3p	6.47×10^{-6}	1.58×10^{-7}	2.44×10^{-2}
hsa-miR-1291	1.96×10^{-5}	5.08×10^{-7}	2.60×10^{-2}
hsa-miR-582-3p	5.22×10^{-6}	1.58×10^{-7}	3.03×10^{-2}
hsa-miR-299-3p	3.90×10^{-5}	1.28×10^{-6}	3.28×10^{-2}
hsa-miR-130a-5p	3.26×10^{-6}	1.13×10^{-7}	3.46×10^{-2}
hsa-miR-629	9.38×10^{-5}	3.69×10^{-6}	3.93×10^{-2}
hsa-miR-483-5p	2.94×10^{-5}	1.22×10^{-6}	4.13×10^{-2}
hsa-miR-200a-5p	2.53×10^{-6}	1.13×10^{-7}	4.46×10^{-2}
hsa-miR-486-5p	2.58×10^{-5}	1.21×10^{-6}	4.68×10^{-2}
hsa-miR-573	1.35×10^{-5}	6.63×10^{-7}	4.89×10^{-2}
hsa-miR-411-5p	3.14×10^{-6}	1.58×10^{-7}	5.00×10^{-2}
hsa-miR-875-5p	3.66×10^{-5}	2.01×10^{-6}	5.48×10^{-2}
hsa-miR-575	1.99×10^{-6}	1.13×10^{-7}	5.68×10^{-2}
hsa-miR-519a-3p	5.54×10^{-5}	3.36×10^{-6}	6.07×10^{-2}
hsa-let-7g-5p	5.27×10^{-6}	3.48×10^{-7}	6.61×10^{-2}
hsa-miR-1208	1.68×10^{-5}	1.17×10^{-6}	6.96×10^{-2}
hsa-miR-409-3p	1.53×10^{-6}	1.13×10^{-7}	7.40×10^{-2}
hsa-miR-938	1.52×10^{-6}	1.13×10^{-7}	7.43×10^{-2}
hsa-miR-1271-5p	5.34×10^{-5}	4.01×10^{-6}	7.51×10^{-2}
hsa-miR-1243	1.29×10^{-6}	1.13×10^{-7}	8.78×10^{-2}
hsa-miR-597	7.41×10^{-6}	6.60×10^{-7}	8.90×10^{-2}
hsa-miR-34a-5p	2.92×10^{-5}	2.90×10^{-6}	9.93×10^{-2}
hsa-miR-221-5p	7.89×10^{-6}	7.87×10^{-7}	9.98×10^{-2}

Supplementary Table 2: Expression of genes affected by miR-200b in T24RC cells. See Supplementary_Table_2

Supplementary Table 3: Expression of genes affected by miR-200b and/or CDDP in T24RC cells. See Supplementary_Table_3

Supplementary Table 4: Sequences of the primers used in this study

	Primer sequence	Product size
miR-200b bisulfite pyrosequencing		
Forward	5′-GGGTTTTATAGAAGTTTTTTTATTTTGGT-3′	159 bp
Reverse	5′-Bio-CAAATATATCCCCTAAACTCCCATAAA-3′	
Sequencing primer	5′-TTGGTTTTTGTTTAATTT-3′	
Sequence to analyze	5′-CGGTGGGCGGTAAGGTGGGGGCG-3′	
miR-200b bisulfite sequencing		
Forward	5′-GGGTTTTATAGAAGTTTTTTTATTTTGGT-3′	345 bp
Reverse	5′-ACAAATAAACAACTCRCCCRTCTCTAA-3′	
miR-200b MSP		
Methylated DNA-specific primers		
Forward	5′-GCGGGGTTCGGGTTTGCGTTATC-3′	126 bp
Reverse	5′-GCCCCACACAAATACGAACTCCCG-3′	
Unmethylated DNA-specific primers		
Forward	5′-GTTTGTGGGGTTTGGGTTTGTGTTATT-3′	131 bp
Reverse	5′-CACCCCACACAAATACAAACTCCCA-3′	
miR-200b ChIP-PCR		
Forward	5′-AAACTCTCCCAGAGACGGG-3′	105 bp
Reverse	5′-GACCTGCAAGGGTGAGCTT-3′	
HAS2 qRT-PCR		
Forward	5′-CTCATCTGTGGAGATGGTAAA-3′	128 bp
Reverse	5′-CCATCCAATATCTTACACTGC-3′	
ZEB1 qRT-PCR		
Forward	5′-GACAAGATATTCCAAAAGAGTAG-3′	136 bp
Reverse	5′-AATGTAATCGCATGTGTTCAATC-3′	
ZEB2 qRT-PCR		
Forward	5′-AAGACATTCCAGAAAAGCAGTT-3′	119 bp
Reverse	5′-TGCTCGATAAGGTGGTGCTT-3′	
TNFSF10 qRT-PCR		
Forward	5′-TGTGTGGCTGTAACTTACGTG-3′	111 bp
Reverse	5′-GTCCCAATAACTGTCATCTTCT-3′	
ACTB qRT-PCR		
Forward	5′-GCCAACCGCGAGAAGATGA-3′	80 bp
Reverse	5′-AGCACAGCCTGGATAGCAAC-3′	

Y = C or T; R = A or G; Bio, biotin.
